# TB co-infection and associated factors among HIV patients attending highly active antiretroviral therapy in Saint Peter's TB Specialized Hospital, Ethiopia: a five years retrospective study

**DOI:** 10.4314/ahs.v24i2.7

**Published:** 2024-06

**Authors:** Dereje Getaw, Fitsum Tigu

**Affiliations:** 1 Department of Biology, Addis Ababa University, Addis Ababa, Ethiopia; 2 Department of Microbial, Cellular and Molecular Biology, Addis Ababa University, Addis Ababa, Ethiopia

**Keywords:** Adherence, ETB, prevalence, TB/HIV co-infection

## Abstract

**Background:**

TB and HIV are the two widely distributed and successful microbial diseases which impose public health problems globally.

**Objectives:**

The study aimed to determine the prevalence and associated factors of TB among people living with HIV (PLHIV).

**Methods:**

A retrospective study was conducted among PLHIV at Saint Peter's TB Specialized Hospital (SPTSH). Study participants were selected by random sampling technique. Logistic regression analyses were employed to determine the associations between dependent and independent variables. P ≤ 0.05 was taken as statistically significant.

**Results:**

The prevalence of TB among PLHIV in the entire study population was 24.6%. The proportion of pulmonary TB (PTB), disseminated TB (DTB) and extra pulmonary TB (EPTB), were 49 (57.6%), 9 (10.6%) and 27 (31.8%), respectively. Logistic regression analysis showed that PLHIV who are non-adhered to ART (AOR = 51.6, 95% CI 24.18 - 387), HAART duration of > 35 months (AOR = 0.39, 95% CI 0.198 - 2.10) and WHO clinical stage IV (AOR = 40.14, 95% CI15.14 - 106.44), were significantly associated with TB/HIV co-infection.

**Conclusions:**

TB co-infection is the major public health issue of PLHIV. Special emphasis is required to reduce the incidence of TB/HIV associated morbidity and mortality among PLHIV

## Introduction

TB and HIV are the two widely distributed and successful microbial diseases whose synergy in pathogenesis has created a significant threat for human health globally. TB is caused by Mycobacterium tuberculosis (MTB). The pathogen is intracellularly invades the alveolar macrophages cells after entering through inhalation of aerosolized bacilli. It is the most common opportunistic infection and a serious health threat for people living with HIV 1. It has a negative impact on immunological activity in HIV patients and increases the progression from HIV inection to acquired immunodeficiency syndrome (AIDS) stage 2. This implies that TB and HIV infections potentiate each other 3.

According to the WHO global TB report, there were big drops in new TB cases worldwide. It falls from 7.1 million to 6.4 million, in 2019 and 2021, respectively. However, contrarily the deaths associated with TB were increased to 1.4 million among HIV negative people and 0.20 million among PLHIV. From the total TB incident cases, 6.7% were PLHIV and African took the highest proportion of TB cases co-infected with HIV 4.

In Ethiopia, from the total notified TB cases in 2020, 41% were bacteriologically confirmed, 27% clinically diagnosed and the remaining 31% were ETB cases. Among the total cases of TB, 40% of deaths were associated with TB and HIV co-infections. There were declining trends of HIV associated TB mortality rates in Ethiopia. This was due to the increased coverage of ART and universal ART (test and treat) policy. However, TB and HIV is still among the highest public health problems of the country^5^.

In most cases, the HIV infected persons with TB have shown the same clinical symptom with that of usual TB patients 6. There are several clinical difficulties to administer HIV positive patients with TB. In developing countries including Ethiopia with a high burden of HIV and TB infections, the problem is more serious. Studies demonstrated that most PLHIVs co-infected with TB have shown an advanced HIV disease and low CD4+ T lymphocyte counts and atypical chest radiograph findings^3^,^7^. The effects of which may appear in any WHO stages of HIV infection and CD4+ T cell counts. However, factors such as low adherence to ART, lower CD4 counts, WHO HIV stages 3 or 4, female sex worker, and older age and being bedridden are increasing the mortality rate of HIV/TB co-infected patients 8-10.

The antiretroviral therapy has a profound effect on reducing the risks of TB/HIV co-infections. However, there are factors associated with immune reconstitution inflammatory syndrome and unmasking of previously subclinical disease, overlapping drug toxicities and drug-drug interactions 6,11. Therefore, the objective of the study was to determine the prevalence of TB and associated factors among PLHIV by analyzing the five years retrospective data of HIV patients attending ART clinic at SPTBSH, Ethiopia.

## Methods

The study was conducted in SPTBSH in Addis Ababa, Ethiopia. The hospital is the principal TB specialized hospital in the country since its inception in 1961. An ART-based retrospective cross- sectional study was carried out to determine the prevalence of TB and associated factors among PLHIVs. A total of 2307 PLHIV patients were registered at ART clinic during January 2015 to December 2019. All HIV patients attending the ART clinic of SPTBSH who were diagnosed as HIV positive and started the treatment with full registration files during the study period were included in the study.

The sample size was calculated using the single population proportion formula, 12. An estimated proportion of TB prevalence among PLHIV was 34% 13, 5% of marginal error (d = 0.05) at 95% of confidence intervals (Zα/2 = 1.96) were used to calculate the actual sample size. Based on the above formula, a calculated value of 345 patients were involved in the study. Five years PLHIV patients' recorded files were identified and 345 study participants were selected by using random sampling.

All socio-demographic data and associated factors were recorded from patients' cards using a prepared checklist adopted from previous studies 14,15. HIV patients with incomplete socio- demographic data such as age, sex, marital status, educational levels, occupation and those who were died were not included in this study. The socio-demographic characteristics such as age, sex, marital status, education, occupation, ART status, HAART duration, CD4 cells count and WHO clinical stage were taken as TB infection among PLHIV patients.

Data were checked for completeness, cleaned and double entered into a computer and analyzed by using the statistical package for social science (SPSS) version 20 software. Descriptive statistics such as frequency and percentages were used to summarize the socio-demographic data. The results were displayed by using tables and graphs. Binary and multiple logistic regression analyses were employed to determine the associations between dependent and independent variables. P ≤0.05 was taken as statistically significant.

## Results

**Figure 1:**
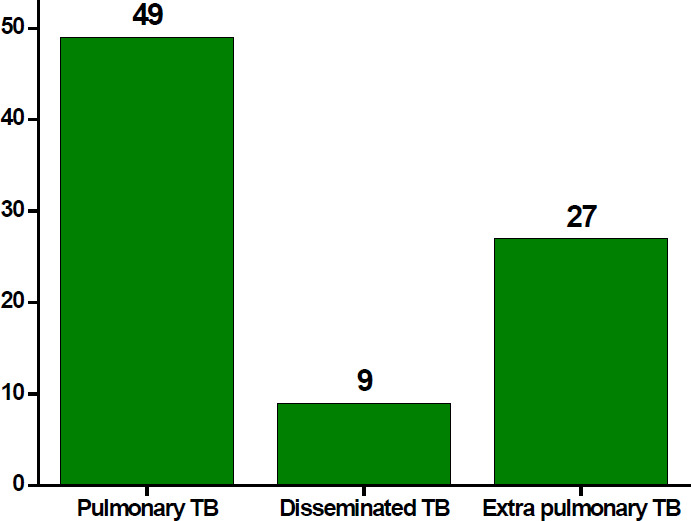
Type of TB

**Figure 2:**
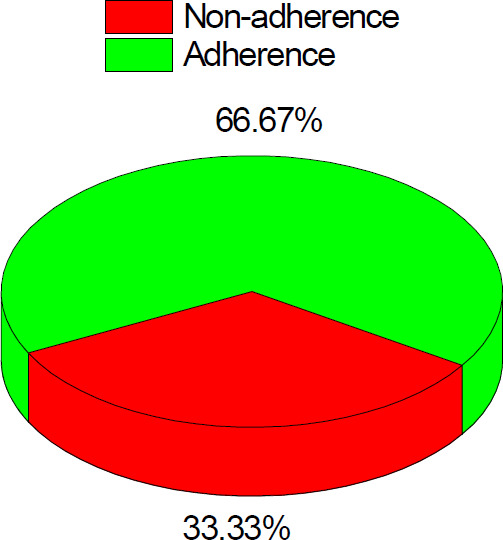
ART status

A total of 345 PLHIV patients (54.8% female and 45.2% male) involved in this study with the major age categories of 31-45 years 144 (41.7%) followed by > 45 years age group accounted for 103 (29.9%). About 166 (48.1%) and 133 (38.6%) were married and single, respectively. Majority 268 (77.7%) of the study participants were literate and 143 (41.4%) of them employed in the private sector (Table 1). From the total PLHIV patients, 85 (24.6%) were TB positive for TB. Of which the proportion of PTB, DTB and EPTB were 49 (57.6%), 9 (10.6%) and 27 (31.8), respectively (Fig. 1). Moreover, based on the ART status of the PLHIV, 224 (64.5%) were adhered to ART (Fig. 2).

**Table 1:** Demographic and clinical profile of study participants in SPTBSH, Ethiopia

Variable	Category	Frequency (%)		

		Total (n=345)	TB Negative (n=260)	TB Positive (n=85)
Age	0-15	20 (5.8)	16 (6.2)	4 (4.7)
	16-30	78 (22.6)	57 (21.9)	21 (24.7)
	31-45	144 (41.7)	110 (42.3)	34 (40.0)
	>45	103 (29.9)	77 (29.6)	26 (30.6)
Sex	Male	156 (45.2)	116 (44.6)	40 (47.1)
	Female	189 (54.8)	144 (55.4)	45 (52.9)
Marital status	Single	133 (38.6)	97 (37.3)	36 (42.4)
	Marriage	166 (77.7)	128 (49.2)	38 (44.7)
	Divorced	46 (13.3)	35 (13.5)	11 (12.9)
	Uneducated	77 (22.3)	15 (5.8)	62 (72.9)
Education	Educated	268 (77.7)	245 (94.2)	23 (27.1)
Occupation	Government	129 (37.1)	101 (38.9)	28 (32.9)
	Private	143 (41.4)	103 (39.6)	40 (47.1)
	Others	73 (21.2)	56 (21.5)	17 (20.0)
WHO stage	Stage one	173 (50.1)	167 (64.2)	6 (7.1)
	Stage two	70 (20.3)	66 (25.4)	4 (4.7)
	Stage three	66 (19.1)	26 (10.0)	40 (47.1)
	Stage four	36 (10.4)	1 (0.4)	35 (41.2)
CD4 T cells	<200	123 (35.7)	50 (19.2)	73 (85.9)
count (cells/mm^3)^	200-349	69 (20)	65 (25.0)	4 (4.7)
	350-500	69 (20)	66 (25.4)	3 (3.5)
	>500	84 (24.4)	79 (30.4)	5 (5.9)

The most frequent WHO clinical stage at the time of HIV diagnosis was stage I comprising 173 (50.1%) of patients followed by stage II comprising 70 (20.3%) while the remaining 102 (29.6%) presented at stages III or IV. About 123 (35.7%) of the patients had baseline CD4 count < 200 cells/mm^3^, whereas 69 (20%) of patients had a baseline CD4 count 200-349 cells/mm^3^. Furthermore, about 69 (20%) of patients had a baseline CD4 count 350-500 cells/mm3 and 84 (24.4%) of patients had a baseline CD4 count > 500 cells/mm^3^.

The bivariate analysis result showed that educational levels, ART status, HAART durations, WHO stage and CD4 cell counts were significantly associated with TB/HIV co-infection (Table 2). HIV patients at WHO clinical stage IV were 6 times more likely to be exposed for TB than those patients who are found at baseline WHO clinical stage I (COR = 6.0, 95% CI 2.0 - 14.3), p < 0.05). Further, HIV patients with CD4 T cells count of < 200 cells/mm^3^ were 27.4 times more likely to be exposed for TB case than those patients with CD4 T cells count > 500 cells/mm^3^ (COR = 27.4, 95% CI 3.47 - 23.43, p < 0.05). Multivariate logistic regression analysis was used to determine TB occurrence among PLHIV (Table 2). ART status, HAART durations, and WHO clinical stage IV (AOR = 40.14; 95% CI 15.14 -106.44) were significant determinants of increased risk of TB co-infection among PLHIV. The study participants who were not adhered to ART were 51.60 times more likely to be exposed for TB cases as compared with those adhered to ART (AOR = 51.6, 95% CI 24.18 - 387), p <0.05. In addition, study participants with HAART duration (months) of less than 35 months were 0.39 times more likely to be exposed for TB case as compared with those whose HAART duration (months) more than 35 months (AOR = 0.39, 95% CI 0.198 - 2.10), p < 0.05. Moreover, the HIV patients with WHO clinical stage IV were 40.14 times more likely to be exposed for TB than patients who were found at baseline WHO clinical stage I (AOR = 401.4, 95% CI 15.14-106.44), p < 0.05.

**Table 2:** Bivariate and multivariate analyses of selected determinant factors of TB-HIV co-infection among HIV patients in SPTBSH

Variable	TB-HIV co-infection		Bivariate analysis		Multivariate analysis	

	Yes N (%)	No N (%)	COR (95% CI),	P value	AOR (95% CI)	P value
Educational status						
Educated	23 (27.1)	206 (79.2)	1	0.011	1	0.720
Uneducated	62 (72.9)	54 (20.8)	1.9 (1.14 - 6.17)		0.836(0.314 - 2.22)	
ART status						
Non-adherence	78 (91.8)	43 (16.5)	41.8 (14.78 - 378)	0.0135	51.6 (24.18 - 387)	0.001
Adherence	1 (0.4)	223 (99.6)	1		1	
HAART duration						
> 35 months	35 (41.2)	121 (46.5)	0.31 (0.19 - 1.987)	0.016	0.39 (0.198 - 2.10)	0.001
< 35 months	8 (9.4)	48 (18.5)	1		1	
WHO stage						
Stage I	6 (7.05)	167 (64.2)	1	0.019	1	
Stage IV	35 (41.2)	1 (0.4)	6.0 (2.0 - 14.3)		40.14 (15.14 - 106.44)	0.001
CD4 T cells count						
>500 cells/mm^3^	5 (5.9)	79 (30.4)	1	0.015	1	0.064
<200 cells/mm^3^	73 (85.9)	50 (19.2)	27.4 (3.47 - 23.43)		0.260(0.62 - 1.08)	

## Discussion

In this study, 345 PLHIV who are attending the antiretroviral therapy clinic were taken into consideration. The aim was to determine the prevalence of TB and its associated factors among PLHIV. The study result revealed that the prevalence of TB/HIV co-infections among the entire study population was found to be 24.6%. This report was in line with other findings in northeastern Ethiopia, 24.3% 15 and higher than the reports from other regions of Ethiopia, it ranges 27.7 - 36.9% 13,14,16 and 39.4% in Mozambique 17. However, this finding was much lower than TB/HIV co-infections reported by other African countries such as Nigeria (13.6%), Uganda (7.2- 15.3%), and Kenya (11.6) 18-20. The variations of TB among PLHIV might be associated with the availability of TB/HIV diagnostic facilities in relation to high TB/HIV burdens in the community.

Several risk factors are reported in the literatures that contributes to the mortality and morbidity of PLHIV in the world. This study also revealed that PLHIV who had no formal education, non- adherence to ART, HAART durations more than 35 months, WHO clinical stage IV and those HIV patients with CD4 T cell counts less than 200 cells/mm^3^ were identified as the most important determinants for TB/HIV co-infections among the HIV patients. A recent study indicated that factors like age of patients, patients living without partners, with no formal education, and low adherence were positively associated with the development of TB among HIV patients 21. Furthermore, use of alcohol, drug toxicity and WHO clinical stages significantly associated with the development of TB in HIV patients 21. The current findings are in line with other health facility-based studies in Ethiopia^14^,^15^.

There are direct relationship between reduction of CD4 T cell count and diseases susceptibility of the patients. The results showed that, patients with CD4 T cell count less than 200 cells/mm^3^ were about 4 times more likely to develop TB as compared to CD4 T cell count more than 200 cells/mm^3^. A similar study in Amhara regional state of Ethiopia also reported that lower CD4 T cell count was the risk factors that the patients to be co-infected more likely than the patients with higher counts of CD4 T cell 13.

Besides, multivariate analysis revealed that adherence status, HAART durations and WHO clinical stages were significantly associated with the development of TB among PLHIV. The study participants with HAART duration of less than 35 months were 0.39 times more likely to be exposed for TB cases as compared with those whose HAART duration was more than 35 months. In line with study results, studies realized that PLHIV who took anti-retroviral drugs less than 12 months had about six times the odds to be infected with TB compared to those taken for ART drugs for more than 36 months 22.

WHO clinical stages are the important determinant factor for the exposure of PLHIV towards TB infection. Our study results showed that HIV patients with WHO clinical stage IV were forty times more likely to be infected with TB than patients who were found at baseline WHO clinical stage I. This finding is consistent with other studies done in public hospitals and health centers in various regions of Ethiopia 13,16,23. Thus, the current study suggests that PLHIV patients who had WHO clinical stage IV might have less immunological efficiencies than WHO clinical stages I.

## Conclusion

In this study, the prevalence of TB among PLHIV was moderate. Among several types of TB diagnosed in SPTBSH, PTB dominantly occurred in HIV patients. The ART adherence, educational status, HAART durations, WHO clinical stages, and CD4+ T cell levels were the major risk factors identified for the infection of TB among PLHIV. Since TB co-infection is the major public health issue for PLHIV, special emphasis is required to reduce the incidence of TB/HIV associated morbidity and mortality among HIV patients.

## Limitations

This study was analyzed the prevalence of TB and factors associated with TB infection among PLHIV. The study was undertaken in a single TB specialized hospital with a limited number of patients in five years retrospective data. Further TB determinants such as viral load, INH and CTX prophylaxis and hemoglobin level with more than ten years retrospective data are required to validate the study findings.

## Data Availability

All the data and materials which are used in this study were included in the manuscript.

## Abbreviations

ART: Antiretroviral therapy
DTB: Disseminated TB
EPTB: Extra pulmonary TB
HAART: Highly active antiretroviral therapy
PLHIV: People living with HIV
PTB: Pulmonary TB
SPTBSH: Saint Peter's TB Specialized Hospital
TB: Tuberculosis
